# Time-dependent effects of hypoxia on mitochondrial dynamics, dysfunction and apoptosis in C8-D1A astrocytes

**DOI:** 10.1515/biol-2025-1353

**Published:** 2026-07-14

**Authors:** Youjuan Chen, Jing Hou, Meiyuan Tian, Erwa Hao, Yaogang Zhang, Dengliang Huang, Tao Zhang, Yanyan Ma

**Affiliations:** Department of Pediatrics, Affiliated Hospital of Qinghai University (Faculty of Clinical Medicine), Xining, 810001, Qinghai Province, China; Central Laboratory, Affiliated Hospital of Qinghai University, Xining, 810001, Qinghai Province, China; Department of Rehabilitation Medicine, Affiliated Hospital of Qinghai University, Xining, 810001, Qinghai Province, China; Qinghai Provincial Women and Children’s Hospital, Xining, 810007, Qinghai Province, China

**Keywords:** astrocyte apoptosis, C8-D1A astrocytes, hypoxia, mitochondria, reactive oxygen species (ROS)

## Abstract

This study aimed to investigate the effects of hypoxia on mitochondrial function in C8-D1A astrocytes and to evaluate its association with apoptosis. Mouse C8-D1A astrocytes were allocated to a normoxia group (21 % O_2_, 5 % CO_2_, 37 °C) or a hypoxia group (1 % O_2_, 5 % CO_2_, 37 °C) and cultured for 24 h, 48 h, or 72 h. Compared with the normoxia group, the hypoxia group demonstrated a significant reduction in mean mitochondrial fluorescence intensity (*p* < 0.01) and a significant increase in mitochondrial ROS levels. Regarding mitochondrial dynamics-related proteins, Mfn1 expression was significantly decreased, and Mfn2 expression was significantly decreased at 72 h (*p* < 0.01). Drp1 expression was significantly increased at all time points (*p* < 0.01), and Fis1 expression was significantly upregulated (*p* < 0.01). The proportion of cells exhibiting reduced mitochondrial membrane potential was significantly higher in the hypoxia group. The percentage of apoptotic cells was significantly increased. TEM analysis revealed structural abnormalities of mitochondria in cells exposed to hypoxia. Hypoxia may promote apoptosis in C8-D1A astrocytes through disruption of mitochondrial morphology, function, and dynamics. These alterations are associated with decreased mitochondrial content and increased ROS production, which may contribute to mitochondrial dysfunction and apoptosis under hypoxic conditions.

## 1 Introduction

High-altitude cerebral edema (HACE) is a severe neurological disorder resulting from rapid exposure to hypobaric hypoxia at high altitude, and is primarily characterized by pronounced neuropsychiatric manifestations, ataxia, and coma [1]. In recent years, the number of individuals traveling to high-altitude areas for tourism, occupational activities, residence, mountaineering, or military operations has increased steadily. As an organ with high oxygen demand, the brain exhibits marked vulnerability to hypoxic conditions. Consequently, rapid ascent to high altitude substantially increases the risk of acute HACE. HACE is a life-threatening condition associated with high morbidity and mortality [2]. Therefore, the identification of effective preventive and therapeutic strategies remains a major focus in high-altitude medicine.

Astrocytes are the most abundant glial cells in the central nervous system (CNS), accounting for approximately 20–60 % of neuroglial cells. These cells perform diverse and essential physiological functions and play a central role in nervous system development, homeostasis, and functional regulation [3]. With advances in the understanding of high-altitude encephalopathy (HAE), evidence indicates that the cerebral vascular basement membrane is ensheathed by astrocytic end-feet [4]. Astrocyte (AS) membranes express multiple ion channels, aquaporins, and amino acid transporters. Under hypoxic conditions, ion transport mediated by these channels is disrupted, leading to upregulation of aquaporin 4 (AQP4), increased aquaporin channel opening, and subsequent intracellular accumulation of water and sodium, thereby contributing to cytotoxic edema in HACE [5]. However, the precise mechanisms underlying astrocyte dysfunction and its regulatory pathways remain incompletely defined.

Mitochondria serve as central regulators of cellular energy production, redox balance, ion homeostasis, and intracellular signaling. Mitochondrial homeostasis and function are maintained through dynamic processes of fusion and fission, which influence cellular fate and functional status [6], 7].

The pathophysiology of HAE is closely associated with oxidative stress and apoptosis, posing significant challenges for prevention and treatment. Nevertheless, the mechanisms by which hypoxia induces astrocyte apoptosis through modulation of mitochondrial function remain unclear. In the present study, C8-D1A astrocytes were used as an *in vitro* model to investigate the effects of hypoxia on mitochondrial morphology, ultrastructure, function, and dynamics, as well as their association with apoptosis. These findings are intended to provide further insight into the pathogenesis of HACE.

## 2 Materials and methods

### 2.1 Experimental materials

The mouse astrocyte cell line C8-D1A (Wuhan Pricella Biotechnology Co., Ltd., CL-0493) was used in this study. Dulbecco’s Modified Eagle Medium (DMEM; Gibco, 12800–017) and fetal bovine serum (FBS; Biological Industries) were used for cell culture. Primary antibodies against mitofusin 1 (mitochondrial fusion protein 1) (Mfn1) (13798-1-AP), dynamin-related protein 1 (Drp1) (12957-1-AP), and mitochondrial fission protein 1 (Fis1) (10956-1-AP) were obtained from Proteintech. Secondary antibodies (goat anti-rabbit/mouse IgG; BOSTER, BA1050) were used for immunodetection. A color pre-stained protein marker and a BCA Protein Assay Kit (Thermo Fisher, 26616, XJ357486) were used for protein quantification. MitoSOX Red Mitochondrial Superoxide Indicator (Invitrogen), a mitochondrial membrane potential assay kit (Solarbio), and MitoTracker^®^ Deep Red FM (Thermo Fisher Scientific) were used for mitochondrial functional analyses.

Instrumentation included a Cytation 5 Cell Imaging Multi-Mode Reader (BioTek), a BD FACSCelesta flow cytometer (BD), an inverted biological microscope (Olympus, CKX41 + DP26), a chemiluminescence gel imaging system (AI600QC, USA), and a tri-gas cell culture incubator (Thermo, 3131, USA).

### 2.2 Experimental methods

#### 2.2.1 Cell culture

C8-D1A mouse astrocytes were cultured at a density of 1 × 10^6^ cells/mL in DMEM supplemented with 10 % fetal bovine serum (FBS) in a humidified incubator maintained at 37 °C with 5 % CO_2_ and 95 % humidity. The culture medium was replaced every 1–2 days. When cell confluence reached approximately 80 %, cells were dissociated with 0.1 % trypsin and passaged. Cells in the logarithmic growth phase with normal morphology were used for subsequent experiments. A total of 1 × 10^6^ C8-D1A astrocytes were assigned to either a normoxia group (21 % O_2_, 5 % CO_2_, 37 °C) or a hypoxia group (1 % O_2_, 5 % CO_2_, 37 °C). Cells were cultured under the respective conditions for 24 h, 48 h, or 72 h.

#### 2.2.2 Western blot (WB) analysis of mitochondrial fusion- and fission-related protein expression in C8-D1A astrocytes in different treatment groups

At each time point, cells from both groups were harvested and lysed with RIPA buffer to extract total protein. Protein concentrations were determined using the BCA assay and normalized accordingly. Following heat denaturation, samples were stored at −80 °C until further analysis. Proteins were separated by 10 % SDS-PAGE and transferred onto polyvinylidene fluoride (PVDF) membranes. Membranes were blocked with 10 % non-fat milk for 1 h at room temperature and incubated overnight at 4 °C with primary antibodies against β-actin (1:1,000), Mfn1 (1:1,000), mitofusin 2 (mitochondrial fusion protein 2) (Mfn2) (1:1,000), Drp1 (1:1,000), and Fis1 (1:1,000) diluted in 1 % BSA. The membranes were washed three times with phosphate buffer saline tween-20 (PBST) (5–6 min per wash) and subsequently incubated with HRP-conjugated goat anti-rabbit/mouse IgG (1:5,000, BOSTER, BA1050) for 1 h at room temperature. After three additional washes with PBST (10–15 min per wash), protein bands were visualized using an ECL chemiluminescence detection system. Band intensities were quantified using ImageJ software (National Institutes of Health, USA).

#### 2.2.3 Detection of mitochondrial ROS in C8-D1A astrocytes

After removal of the culture medium, cells were washed with 2 mL of 0.01 mol/L phosphate buffer saline (PBS) and incubated with 5 μmol/L MitoSOX Red working solution at 37 °C in 5 % CO_2_ for 30 min in the dark. Cells were gently washed twice with PBS and then incubated with 10 μg/mL Hoechst working solution at 37 °C for 15 min in the dark. After two additional washes with PBS, cells were fixed with paraformaldehyde at room temperature for 10 min in the dark and washed again with PBS. Mitochondrial reactive oxygen species (ROS) fluorescence intensity was measured using a Cytation 5 Cell Imaging Multi-Mode Reader.

#### 2.2.4 Detection of mitochondrial membrane potential in C8-D1A astrocytes

At each time point, approximately 1 × 10^6^ cells were collected and transferred into flow cytometry tubes. Cells were incubated with 0.5 mL of freshly prepared JC-1 staining working solution for 15 min in the dark. Subsequently, 2 mL of JC-1 staining buffer was added, and samples were gently mixed. Cells were centrifuged at 450 × *g* for 5 min (rotor radius: 10.5 cm), and the supernatant was discarded. After washing with 1 mL of 0.01 mol/L PBS, cells were resuspended in 0.3 mL of PBS and analyzed by flow cytometry. Mitochondrial membrane potential was analyzed using a BD FACSCelesta flow cytometer equipped with 488 nm laser. For JC-1 staining, fluorescence was detected using 488 nm excitation with emission filters at 530/30 nm (green, monomer) and 585/42 nm (red, aggregate).

#### 2.2.5 Mitochondrial labeling with MitoTracker^®^ Deep Red FM in C8-D1A astrocytes

C8-D1A astrocytes in good condition were seeded into culture dishes at a density of 5 × 10^4^ cells/mL. After incubation for 24 h, 48 h, and 72 h, the culture medium was removed. Cells were incubated with medium containing 50 ng/mL MitoTracker^®^ Deep Red FM at 37 °C for 30 min. The staining solution was discarded, and cells were washed with 2 mL of 0.01 mol/L PBS. Mean mitochondrial fluorescence intensity was determined using a Cytation 5 Cell Imaging Multi-Mode Reader.

#### 2.2.6 Detection of apoptosis in C8-D1A astrocytes

At each time point, approximately 1 × 10^6^ cells were collected, washed with PBS, and centrifuged. The supernatant was discarded, and cells were resuspended in staining solution containing Annexin V (50 μL) and propidium iodide (PI, 100 μL). Samples were incubated at room temperature for 15–20 min in the dark and analyzed immediately by flow cytometry. Apoptosis was analyzed using a BD FACSCelesta flow cytometer equipped with 488 nm laser For Annexin V/PI staining, fluorescence was detected using 488 nm excitation with emission filters at 530/30 nm (FITC, Annexin V) and 585/42 nm (PI). Data acquisition was performed using BD FACSDiva software, and a minimum of 10,000 events were collected per sample.

#### 2.2.7 Transmission electron microscopy (TEM) analysis of mitochondria in C8-D1A astrocytes

At 24 h, 48 h, and 72 h, the culture medium was removed from the dishes in each group. Subsequently, 5 mL of pre-chilled fixative (2.5 % glutaraldehyde in 0.1 M phosphate buffer, pH 7.4) was added to each dish, and samples were fixed undisturbed for 1 h. Cells were collected using a cell scraper and transferred to a 15 mL centrifuge tube. Following centrifugation at 600 rpm for 5 min, the supernatant was removed, leaving approximately 1.0 mL of residual volume. The cell pellet was gently resuspended and transferred to an Eppendorf (EP) tube for an additional 1 h of fixation. After removal of the supernatant, 1 mL of pre-chilled fixative was added, and the tube was sealed with Parafilm. Post-fixation was performed with 1 % osmium tetroxide in 0.1 M phosphate buffer for 1 h. The specimens were subsequently submitted to Wuhan Luochuang Biotechnology Co., Ltd. for analysis.

### 2.3 Statistical methods

All data were derived from at least three independent experiments. Normality of distribution was assessed using the Shapiro-Wilk test. Measurement data with a normal distribution are presented as the mean ± standard deviation (SD). Intergroup comparisons were conducted using two-way analysis of variance (two-way ANOVA), followed by simple effects analysis where appropriate. The Šidák correction was applied for planned pairwise comparisons between normoxia and hypoxia groups at each time point, while Tukey’s honestly significant difference (HSD) test was used for post hoc pairwise comparisons among multiple time points within the same treatment group. Statistical analyses were performed using GraphPad Prism 9.5 software. A two-tailed *p* value < 0.05 was considered to indicate statistical significance.

## 3 Results

### 3.1 Hypoxia reduces the mean mitochondrial fluorescence intensity in C8-D1A astrocytes

**Figure 1:**
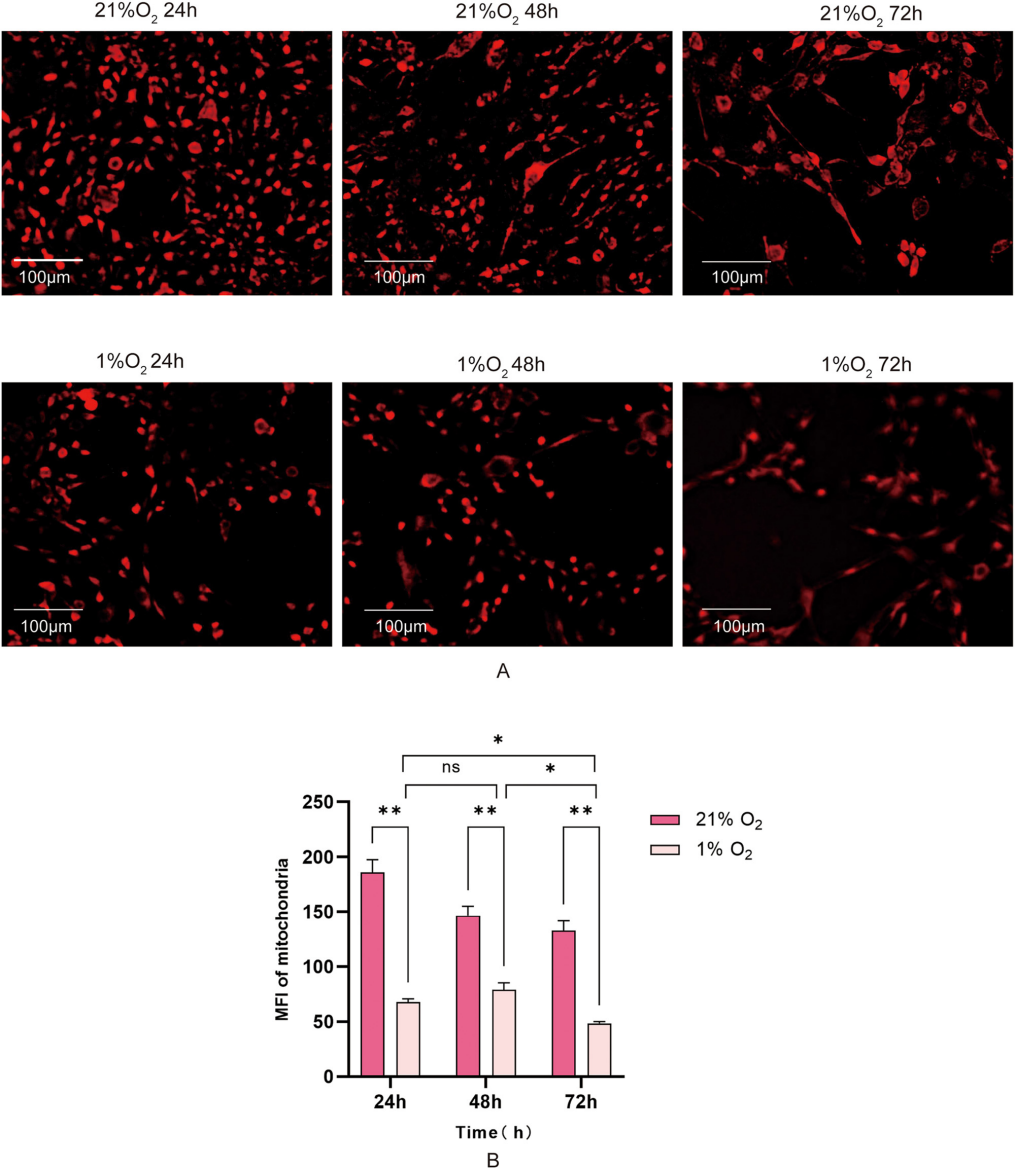
Effects of hypoxia and exposure duration on mean mitochondrial fluorescence intensity in C8-D1A astrocytes (*n* = 3). Data are presented as mean ± SD; statistical significance was determined by two-way ANOVA followed by Šidák correction for intergroup comparisons and Tukey’s test for intragroup comparisons; *p < 0.05, **p < 0.01, ***p < 0.001. A: Representative images of mitochondrial fluorescence in C8-D1A astrocytes cultured under normoxic (21 % O_2_) and hypoxic (1 % O_2_) conditions at indicated time points; B: Quantitative analysis of relative mean mitochondrial fluorescence intensity under different oxygen concentrations and exposure durations.

C8-D1A astrocytes were cultured under normoxic (21 % O_2_) or hypoxic (1 % O_2_) conditions for different durations. Mean mitochondrial fluorescence intensity was assessed using an inverted fluorescence microscope (Figure 1A). Compared with the normoxia group, cells exposed to hypoxia for 24 h, 48 h, and 72 h exhibited significantly lower relative mean mitochondrial fluorescence intensity at the corresponding time points (hypoxia: 67.96 ± 3.05, 78.96 ± 6.54, and 48.40 ± 1.69 vs. normoxia: 185.88 ± 11.71, 146.47 ± 8.57, and 133.03 ± 8.99; *p* < 0.01). Furthermore, within the hypoxia group, fluorescence intensity at 72 h was significantly lower than that at 24 h and 48 h (*p* < 0.05; Figure 1B).

### 3.2 Hypoxia significantly increases the mean mitochondrial ROS fluorescence intensity in C8-D1A astrocytes

**Figure 2:**
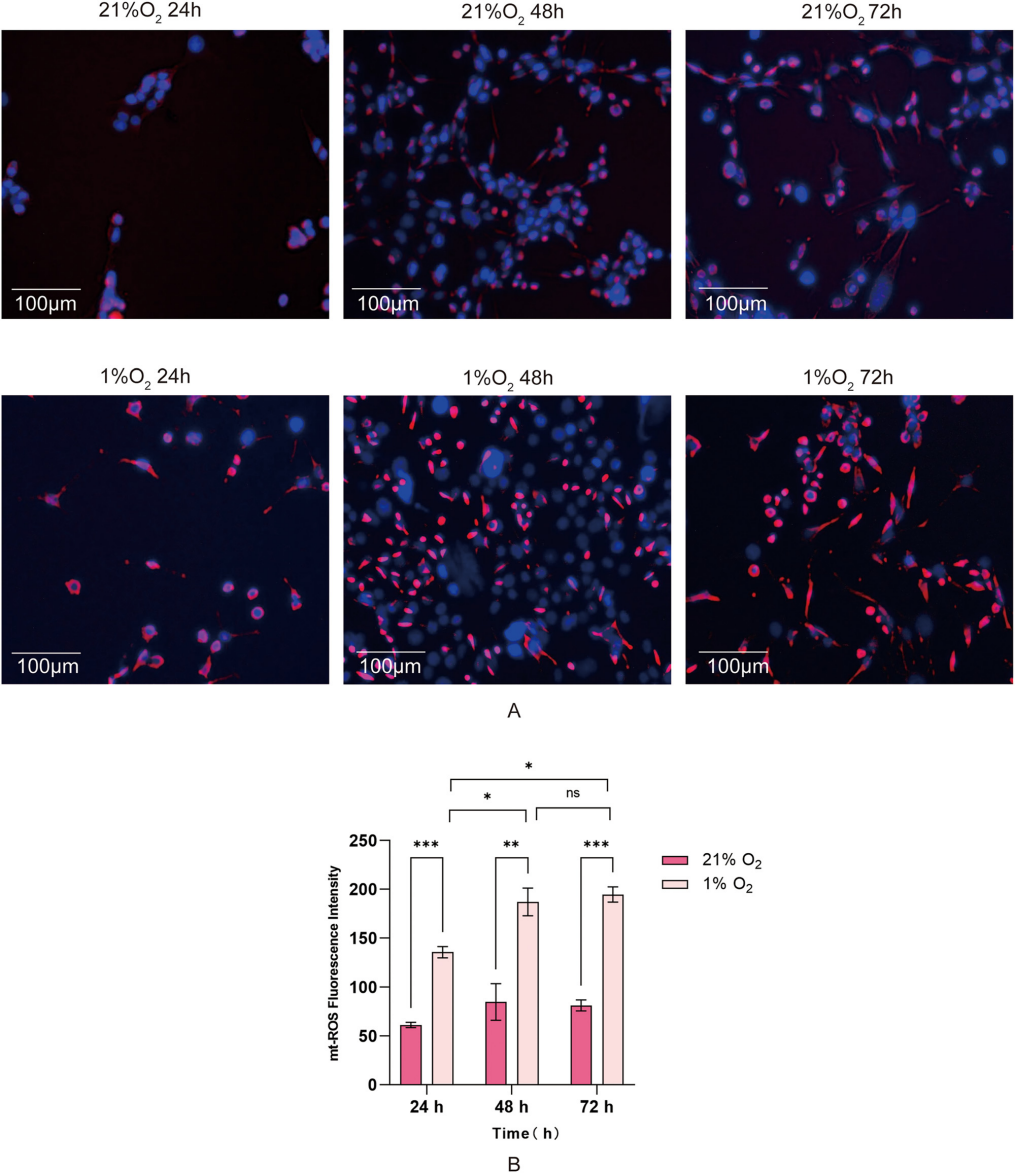
Effects of hypoxia and exposure duration on mitochondrial ROS levels in C8-D1A astrocytes (*n* = 3). Data are presented as mean ± SD; statistical significance was determined by two-way ANOVA followed by Šidák correction for intergroup comparisons and Tukey’s test for intragroup comparisons; *p < 0.05, **p < 0.01, ***p < 0.001. A: Representative images demonstrating mitochondrial ROS levels detected by MitoSOX Red staining under normoxic and hypoxic conditions at indicated time points; B: Quantitative analysis of relative mitochondrial ROS fluorescence intensity under different oxygen concentrations and exposure durations.

Mitochondrial ROS levels were evaluated using MitoSOX Red staining in C8-D1A astrocytes cultured under normoxic or hypoxic conditions. Mean mitochondrial ROS fluorescence intensity was observed using an inverted fluorescence microscope (Figure 2A). Cells cultured under hypoxia for 24 h, 48 h, and 72 h demonstrated significantly higher mitochondrial ROS fluorescence intensity compared with the normoxia group at the corresponding time points (hypoxia: 986.33 ± 13.05, 1,267.66 ± 52.27, and 1,003.33 ± 14.04 vs. normoxia: 878.66 ± 20.03, 1,003.33 ± 14.04, and 937.00 ± 19.07; *p* < 0.05). With increasing duration of hypoxic exposure, mitochondrial ROS levels demonstrated an overall upward trend, reaching the highest level at 72 h (Figure 2B).

### 3.3 Prolonged hypoxia down-regulates Mfn1 and Mfn2 expression and up-regulates Drp1 and Fis1 expression in mitochondria

**Figure 3:**
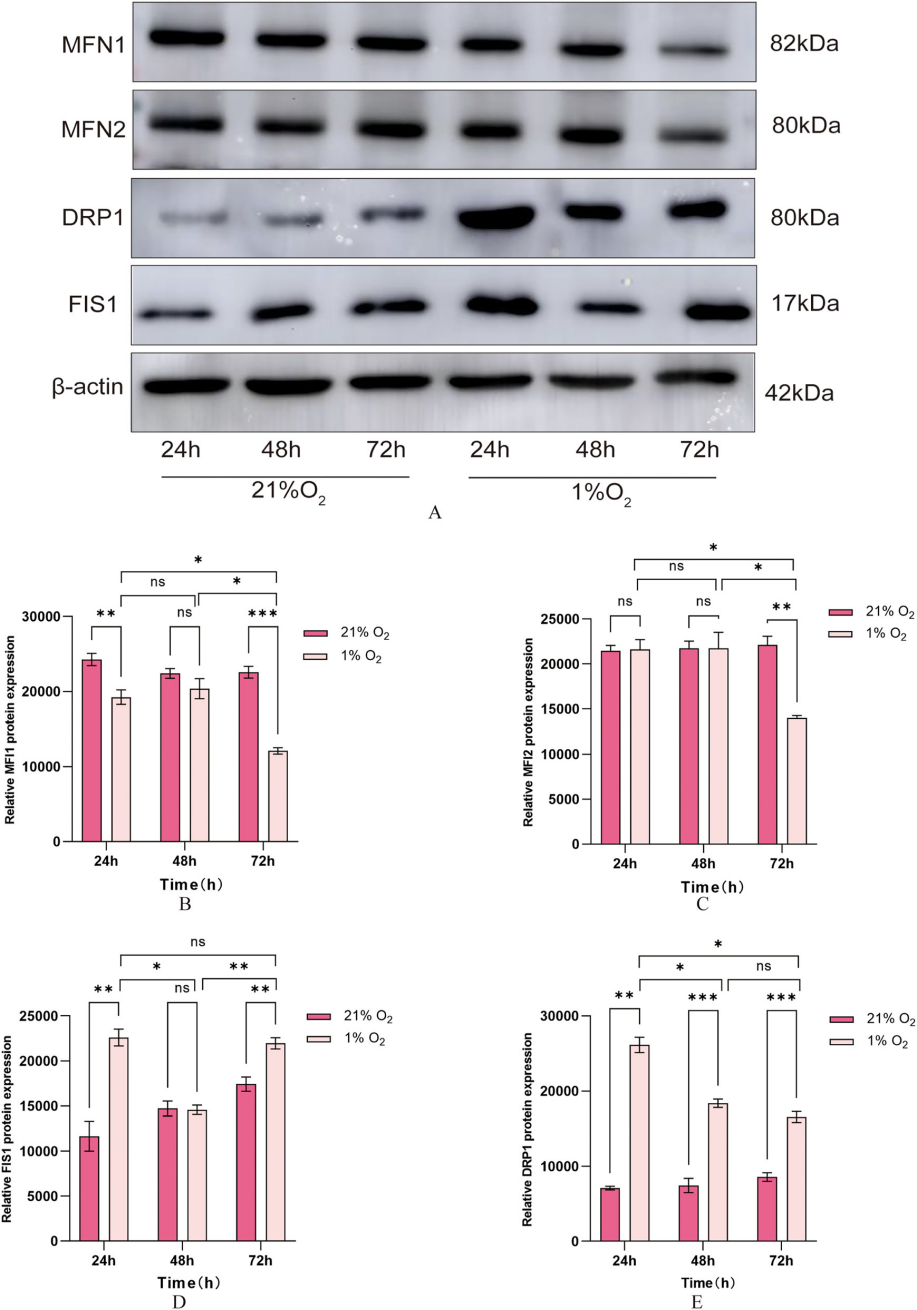
Effects of hypoxia and exposure duration on mitochondrial fusion- and fission-related protein expression in C8-D1A astrocytes (*n* = 3). Data are presented as mean ± SD; statistical significance was determined by two-way ANOVA followed by Šidák correction for intergroup comparisons and Tukey’s test for intragroup comparisons; *p < 0.05, **p < 0.01, ***p < 0.001. A: Western blot analysis and relative expression levels of Mfn1, Mfn2, Drp1, and Fis1 under normoxic and hypoxic conditions at indicated time points; B: Relative expression level of Mfn1 protein at different oxygen concentrations and time points; C: Relative expression level of Mfn2 protein at different oxygen concentrations and time points; D: Relative expression level of Drp1 protein at different oxygen concentrations and time points; E: Relative expression level of Fis1 protein at different oxygen concentrations and time points.

Western blot results are presented in Figure 3A. In the hypoxia group, the relative expression levels of Mfn1 at 24 h and 72 h were 19,241.55 ± 950.73 and 12,092.89 ± 435.19, respectively, compared with 24,254.24 ± 810.82 and 22,557.52 ± 779.43 in the normoxia group. At 72 h, Mfn2 expression was 14,010.64 ± 239.46 in the hypoxia group and 22,129.59 ± 951.82 in the normoxia group. Expression levels of the mitochondrial fusion proteins Mfn1 and Mfn2 were significantly lower in the hypoxia group than in the normoxia group (*p* < 0.05; Figures 3B and C). In contrast, the relative expression levels of the mitochondrial fission protein Drp1 in the hypoxia group at 24 h, 48 h, and 72 h were 26,128.35 ± 1,019.72, 18,365.22 ± 554.46, and 16,543.02 ± 730.57, respectively, compared with 7,089.37 ± 251.12, 7,438.02 ± 940.45, and 8,565.68 ± 578.03 in the normoxia group (*p* < 0.01). FIS1 expression at 24 h and 72 h was 22,599.44 ± 932.82 and 21,961.27 ± 625.47 in the hypoxia group, compared with 11,647.44 ± 1,652.59 and 17,424.38 ± 789.49 in the normoxia group. Both Drp1 and Fis1 expression levels were significantly elevated in the hypoxia group (*p* < 0.01; Figures 3D and E). Moreover, Mfn1 and Mfn2 expression levels at 72 h were significantly lower than those at 24 h within the hypoxia group (*p* < 0.05; Figures 3B and C).

### 3.4 Hypoxia promotes the reduction of mitochondrial membrane potential in C8-D1A astrocytes

**Figure 4:**
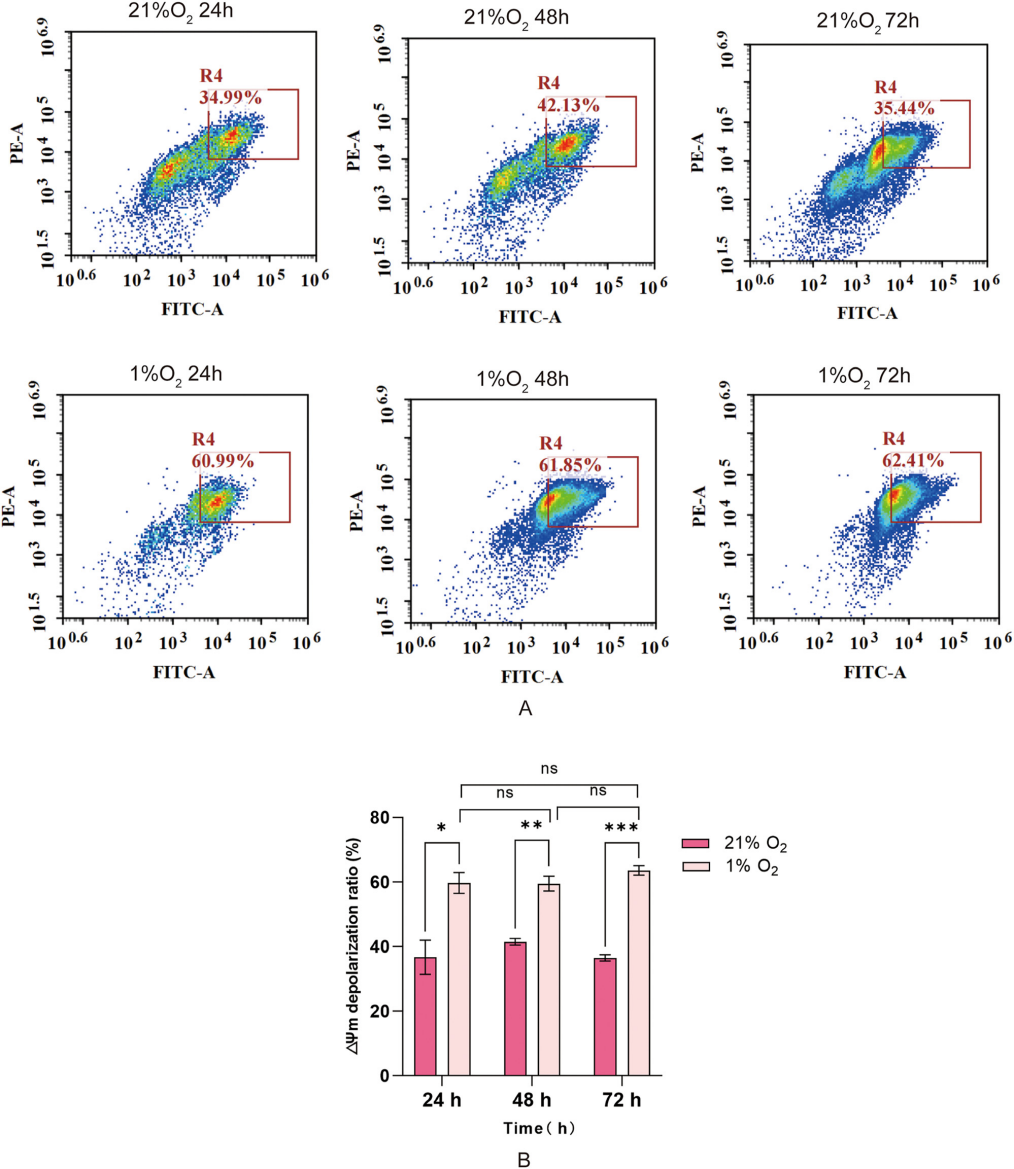
Effects of hypoxia and exposure duration on mitochondrial membrane potential in C8-D1A astrocytes (*n* = 3). Data are presented as mean ± SD; statistical significance was determined by two-way ANOVA followed by Šidák correction for intergroup comparisons and Tukey’s test for intragroup comparisons; *p < 0.05, **p < 0.01, ***p < 0.001. A: Flow cytometric analysis of mitochondrial membrane potential under normoxic and hypoxic conditions at indicated time points; B: Quantitative analysis of the percentage of cells with decreased mitochondrial membrane potential.

Mitochondrial membrane potential was evaluated by flow cytometry (Figure 4A). The P4 gate represents the cell population with depolarized mitochondrial membrane potential. The proportions of cells with reduced mitochondrial membrane potential after 24 h, 48 h, and 72 h of hypoxia were 59.69 ± 3.24 %, 59.48 ± 2.28 %, and 63.59 ± 1.43 %, respectively. These values were significantly higher than those in the normoxia group (36.70 ± 5.23 %, *p* < 0.05; 41.43 ± 1.03 %, *p* < 0.01; 36.53 ± 0.96 %, *p* < 0.001; Figure 4B).

### 3.5 Hypoxia damages mitochondrial structure in C8-D1A astrocytes

**Figure 5:**
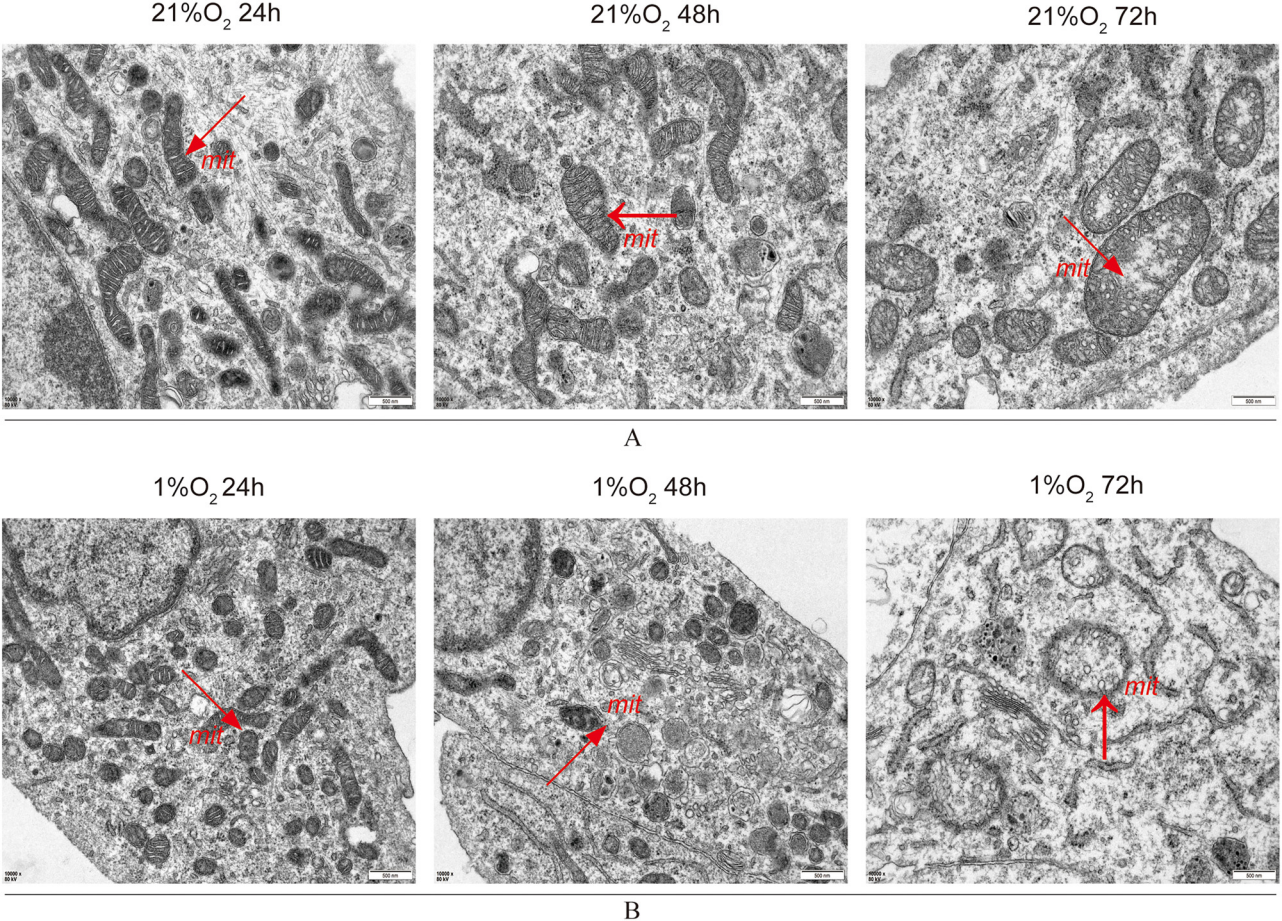
Ultrastructural alterations of mitochondria in C8-D1A astrocytes under normoxic and hypoxic conditions. A: TEM images of mitochondria in C8-D1A astrocytes cultured under normoxia at indicated time points; B: TEM images of mitochondria in C8-D1A astrocytes cultured under hypoxia at indicated time points (mit = mitochondrion).

TEM findings are presented in Figure 5. In the hypoxia group, mitochondria exhibited varying degrees of morphological abnormalities after 24 h, including reduced volume, partial disruption and fragmentation of cristae, and increased membrane density (Figure 5D–F). In contrast, mitochondria in the normoxia group displayed normal ultrastructural features characterized by larger volume, well-organized cristae, and relatively low membrane density (Figure 5A–C). With prolonged hypoxic exposure, mitochondrial structural disorganization became more pronounced, particularly at 72 h, as evidenced by marked cristae fragmentation, heterogeneous mitochondrial size, and increased membrane density.

### 3.6 Hypoxia increases the apoptosis rate of C8-D1A astrocytes

**Figure 6:**
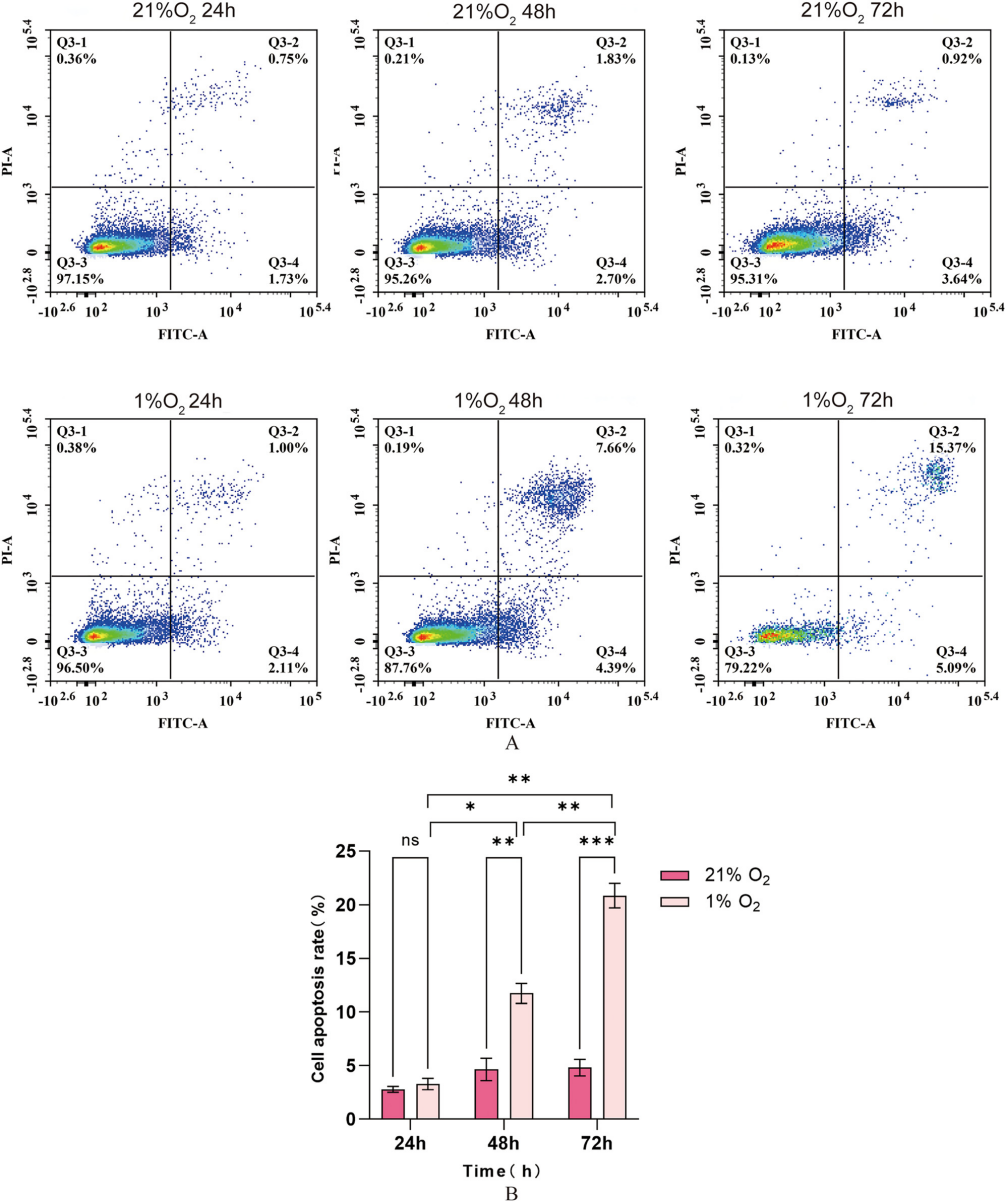
Effects of hypoxia and exposure duration on apoptosis in C8-D1A astrocytes (*n* = 3). Data are presented as mean ± SD; statistical significance was determined by two-way ANOVA followed by Šidák correction for intergroup comparisons and Tukey’s test for intragroup comparisons; *p < 0.05, **p < 0.01, ***p < 0.001. A: Flow cytometric analysis of apoptosis in C8-D1A astrocytes under normoxic and hypoxic conditions at indicated time points; B: Quantitative analysis of relative apoptosis rates under different oxygen concentrations and exposure durations.

Apoptosis was assessed by flow cytometry (Figure 6A). The Q3-3 gate represents the target cell population, while Q3-2 and Q3-4 correspond to apoptotic cell populations. The apoptosis rates in the hypoxia group at 48 h and 72 h were 11.73 ± 0.92 % and 20.87 ± 1.14 %, respectively, both significantly higher than those in the normoxia group (4.63 ± 1.04 %, *p* < 0.01; 4.81 ± 0.76 %, *p* < 0.001; Figure 6B). With increasing duration of hypoxic exposure, the apoptosis rate progressively increased and reached its highest level at 72 h.

## 4 Discussion

HAE is a central nervous system disorder primarily attributable to acute exposure to a hypobaric hypoxic environment. Its principal clinical manifestations include high-altitude headache (HAH), acute mountain sickness (AMS), and high-altitude cerebral edema (HACE) [8]. Approximately 26 % of the global land surface comprises high-altitude regions. In China, areas located above 3,000 m account for 26.8 % of the national territory. With ongoing development of high-altitude regions, increasing numbers of individuals are entering these environments for occupational, residential, and recreational purposes, resulting in a rising incidence of high-altitude–related illnesses. Among these conditions, HACE represents the most severe form of HAE and is characterized by severe headache, vomiting, ataxia, and progressive impairment of consciousness [9]. According to the Lake Louise diagnostic criteria, HACE is classified into vasogenic HACE, which occurs without preceding AMS but presents with neuropsychiatric symptoms and ataxia, and cytotoxic HACE, which develops secondary to AMS and is accompanied by altered mental status or ataxia [10]. In advanced stages, vasogenic and cytotoxic forms may coexist and interact, contributing to disease progression.

Astrocytes constitute the most abundant glial cell population in the CNS and perform multiple essential functions, including structural support, maintenance and regulation of the blood–brain barrier, participation in neurodevelopment and post-injury repair, modulation of neuroimmune responses, neurotransmitter metabolism, and regulation of cerebral metabolism and ion homeostasis [11], 12]. Substantial evidence indicates that astrocytes are involved in the pathogenesis of various neurological disorders, including neurodegenerative diseases, stroke, and hypoxic brain injury [2]. The cerebral vascular basement membrane is ensheathed by astrocytic end-feet, and astrocytic membranes express diverse ion channels, aquaporins such as AQP4, and amino acid transporters [5].

Under physiological conditions, cellular uptake of ions and neurotransmitters increases intracellular osmotic pressure, leading to transient water influx and reversible cell swelling. Ion channels implicated in HACE pathogenesis include volume-regulated anion channels (VRACs), chloride channels (Cl^−^), and inwardly rectifying potassium channel 4.1 (Kir4.1). Rapid exposure to hypoxia disrupts transmembrane ion exchange, resulting in upregulation of AQP4, excessive aquaporin activation, and intracellular retention of water and sodium, thereby contributing to cytotoxic HACE [13], 14].

Concurrent astrocytic degeneration, necrosis, and swelling of astrocytic processes may compromise tight junction integrity within the basement membrane, impair barrier function, and exacerbate vasogenic edema. At advanced stages, vasogenic and cytotoxic mechanisms may potentiate one another, increasing mortality risk [9]. Nevertheless, the astrocyte-mediated molecular mechanisms underlying HACE remain incompletely understood.

Mitochondria are central regulators of cellular energy metabolism and signal transduction. Through maintenance of membrane potential, regulation of the internal microenvironment, and dynamic fusion and fission processes, mitochondria preserve structural and functional homeostasis, thereby influencing cellular survival and fate [15]. Mitochondrial fusion is mediated by mitofusin 1 and mitofusin 2 (Mfn1/Mfn2), which enhance mitochondrial functional capacity under conditions of oxidative stress or increased metabolic demand. In contrast, mitochondrial fission is primarily driven by dynamin-related protein 1 (Drp1), which interacts with mitochondrial fission protein 1 (Fis1) to promote mitochondrial division. Upon stimulation, Drp1 undergoes phosphorylation and translocates to the outer mitochondrial membrane, where it recruits FIS1 to initiate fission.

Excessive fission may increase ROS production and modulate processes such as energy metabolism, ion homeostasis, apoptosis, autophagy, and immune activation [6]. Mitochondrial abnormalities, including morphological alterations, reduced membrane potential, and excessive ROS generation, have been closely associated with astrocytic dysfunction. Dysregulated astrocytic signaling may disrupt synaptic and neurotransmitter homeostasis, potentially contributing to cognitive impairment [16], 17].

The present study examined the “hypoxia–mitochondrial dysfunction–apoptosis” axis in C8-D1A astrocytes to clarify how hypoxia may induce apoptosis through mitochondrial impairment, thereby providing insight into the pathogenesis of HACE.

Significant alterations in mitochondrial function were observed under hypoxic conditions. Mean mitochondrial fluorescence intensity was markedly reduced in hypoxia-exposed astrocytes compared with normoxic controls, indicating decreased mitochondrial content or functional integrity. ROS, recognized as by-products of mitochondrial dysfunction, play a critical role in oxidative stress and inflammatory signaling [17]. MitoSOX Red staining demonstrated significantly elevated mitochondrial ROS levels in the hypoxia group, with progressive increases over time and peak levels at 72 h. Excessive ROS accumulation may induce oxidative damage to proteins, deoxyribonucleic acid (DNA), and lipids, resulting in mitochondrial swelling, reduced membrane potential, opening of the mitochondrial permeability transition pore, and release of pro-apoptotic factors such as cytochrome *c*, thereby compromising cell viability [18].

In addition to mitochondrial dynamics and ROS accumulation, hypoxia-induced cellular stress also involves plasma membrane perturbations. Under hypoxic conditions, altered mechanotransduction signaling and compromised membrane repair mechanisms may contribute to cellular injury. Recent evidence demonstrates that plasma membrane disruptions (PMDs) initiate downstream mechanotransduction responses, and impaired membrane repair capacity can exacerbate cellular vulnerability to stress [19]. In the context of hypoxia, such membrane stress may interact with mitochondrial dysfunction by disrupting calcium homeostasis and ion signaling, thereby amplifying apoptotic cascades. Although the present study did not directly assess membrane integrity or mechanotransduction pathways, these processes represent important complementary mechanisms that warrant investigation in future studies. Calcium signaling represents a crucial but previously underemphasized component of hypoxia-induced mitochondrial dysfunction in astrocytes.

Under hypoxic conditions, disrupted calcium homeostasis contributes to multiple aspects of mitochondrial injury. Elevated cytosolic calcium concentrations promote mitochondrial calcium overload, which triggers opening of the mitochondrial permeability transition pore (mPTP), dissipates mitochondrial membrane potential, and amplifies ROS production [20], 21]. Furthermore, calcium-dependent activation of calpains and other proteases can directly damage mitochondrial structural proteins, while calcium-mediated signaling pathways regulate the activity of Drp1 and other fission proteins, thereby promoting mitochondrial fragmentation. In astrocytes, calcium dysregulation under hypoxia has been shown to impair glutamate uptake, disrupt neurotransmitter homeostasis, and exacerbate excitotoxicity, all of which converge on mitochondrial dysfunction and apoptotic signaling [20]. The absence of direct calcium measurements in the present study represents a limitation, and future investigations should incorporate dynamic calcium imaging to comprehensively characterize the hypoxia-calcium-mitochondria axis in astrocytes.

Mitochondrial membrane potential, generated during oxidative phosphorylation, constitutes the electrochemical gradient required for ATP synthesis. Under physiological conditions, this potential remains stable; its dissipation is indicative of mitochondrial dysfunction and cellular injury [22]. Flow cytometric analysis demonstrated significant depolarization of mitochondrial membrane potential in hypoxia-exposed astrocytes, particularly at 72 h, indicating progressive mitochondrial impairment. The interplay between plasma membrane stress and mitochondrial dysfunction under hypoxic conditions represents a critical but underexplored aspect of cellular injury. Hypoxia-induced membrane stress can trigger aberrant calcium influx, which overloads mitochondria and promotes opening of the mitochondrial permeability transition pore (mPTP). This, in turn, dissipates mitochondrial membrane potential, amplifies ROS production, and releases pro-apoptotic factors such as cytochrome *c* [21]. Furthermore, impaired mechanotransduction signaling may compromise the cell’s ability to sense and respond to environmental stress, thereby reducing adaptive capacity and promoting cell death. These membrane-mitochondria interactions suggest that mitochondrial dysfunction in hypoxia-exposed astrocytes may be partially mediated by upstream membrane perturbations, highlighting the need for multimodal assessment of cellular injury in future investigations.

Mitochondria are dynamic double-membrane organelles in eukaryotic cells that play essential roles in cellular differentiation, proliferation, and death. Their functional capacity depends on structural integrity, which is regulated by mitochondrial dynamics, including fusion and fission processes. Previous studies have demonstrated that coordinated fusion and fission are indispensable for maintaining mitochondrial homeostasis [23]. The equilibrium between these processes determines mitochondrial morphology, number, and intracellular distribution, allowing mitochondria to localize to regions of high metabolic demand. This dynamic balance supports physiological cellular functions, modulates complex signaling pathways such as metabolic regulation, and influences cellular proliferation and apoptosis [15].

To further clarify the impact of hypoxia on mitochondrial dynamics in C8-D1A astrocytes, the expression of fusion- and fission-related proteins was evaluated. Under hypoxic conditions, the expression levels of the fusion proteins Mfn1 and Mfn2 were significantly reduced, whereas the fission proteins Drp1 and Fis1 were significantly upregulated. These alterations indicate a shift toward enhanced mitochondrial fission and suppressed fusion, favoring mitochondrial fragmentation. Consistent with these molecular findings, TEM revealed marked ultrastructural abnormalities in hypoxia-exposed astrocytes, including reduced mitochondrial volume, cristae disruption, structural disorganization, and increased membrane density. The TEM findings of mitochondrial fragmentation, cristae disruption, and increased membrane density under hypoxia reflect not only morphological alterations but also underlying structural fragility and impaired membrane resilience. The disruption of cristae architecture compromises the efficiency of oxidative phosphorylation and ATP synthesis, while increased membrane density may indicate altered lipid composition or protein aggregation, both of which impair membrane fluidity and function. These structural changes render mitochondria more susceptible to permeability transition and reduce their capacity to maintain membrane potential under stress. The observed structural fragility likely contributes to the progressive functional decline documented by reduced MitoTracker fluorescence, elevated ROS levels, and depolarized membrane potential, collectively creating a vicious cycle of mitochondrial deterioration that ultimately promotes apoptotic cell death. Previous pathological studies of HACE have reported prominent astrocyte swelling and necrosis, which contribute to disease progression [24].

Flow cytometric analysis in the present study demonstrated no significant increase in apoptosis after 24 h of hypoxic exposure; however, apoptosis rates increased progressively with prolonged hypoxia, reaching the highest level at 72 h. The lack of significant increase in apoptosis at 24 h of hypoxic exposure may reflect the temporal dynamics of the mitochondrial fission-ROS-apoptosis axis. At this early time point, mitochondrial dysfunction is already evident (reduced membrane potential, increased ROS, altered dynamics proteins), but the cumulative damage may not yet have reached the threshold required to trigger execution-phase apoptotic cascades. The observed upregulation of Drp1 and Fis1 at 24 h indicates active mitochondrial fragmentation, which may initially serve an adaptive quality control function through mitophagic clearance of damaged mitochondria. However, as hypoxic exposure extends to 48 h and 72 h, the balance shifts from adaptive fission toward maladaptive fragmentation, excessive ROS overwhelms antioxidant defenses, and the mitochondrial membrane potential collapse becomes irreversible, ultimately activating caspase-dependent apoptotic pathways. This time-dependent progression suggests that astrocytes possess a limited window of adaptive capacity under hypoxia, beyond which mitochondrial injury becomes irreversible and apoptotic cell death ensues. Although these findings indicate an association between mitochondrial dysfunction and apoptosis, whether mitochondrial fragmentation directly mediates apoptotic signaling requires further investigation.

## 5 Conclusions

In summary, hypoxia markedly impairs mitochondrial function in C8-D1A astrocytes. Disruption of mitochondrial membrane potential and imbalance in fusion–fission dynamics may promote excessive mitochondrial ROS generation, resulting in structural damage, altered membrane density, and compromised mitochondrial integrity, ultimately reducing mitochondrial content. Through these coordinated effects on mitochondrial structure, function, and dynamics, hypoxia may facilitate apoptosis in C8-D1A astrocytes.

Several limitations should be acknowledged. First, pharmacological or genetic interventions targeting mitochondrial fusion or fission were not performed; therefore, the specific mechanistic pathways remain incompletely defined. Second, extended time points were not included, limiting the assessment of the long-term effects of chronic hypoxia on mitochondrial and cellular function. Third, the present study primarily employed mitochondrial-centric endpoints to assess hypoxia-induced cellular injury. While these parameters provide valuable insights into mitochondrial dysfunction, stress-induced cellular dysfunction involves multiple interconnected pathways, including plasma membrane perturbation, calcium dysregulation, and impaired mechanotransduction signaling. Multimodal injury assessment approaches that integrate mitochondrial function, membrane integrity, calcium dynamics, and cellular biomechanics would provide a more comprehensive understanding of hypoxia-induced astrocyte injury and are recommended for future investigations [25]. Meanwhile, this study has several limitations: the experiments were conducted using only a single cell line, *in vivo* validation was lacking, and mitochondrial ATP production was not detected. Future investigations should further delineate the molecular mechanisms linking hypoxia-induced mitochondrial dysfunction to astrocyte apoptosis and establish appropriate disease models to enhance translational relevance and improve the understanding of HAE pathogenesis.
